# Algicidal Monoterpenes Against Toxin-Producing *Microcystis aeruginosa* with Reduced Toxicity Toward *Chlorella sorokiniana*: In Vitro, Molecular Docking, and ADMET Study

**DOI:** 10.3390/toxins18060258

**Published:** 2026-06-05

**Authors:** El Mehdi Darrag, Yasser Essadki, Saad Zekri, Halima Chernane, Abderrahmane Romane, Ismail Hdoufane, Driss Cherqaoui, Brahim Oudra, Abdelilah Meddich, Vitor Vasconcelos, Abdelaziz Baçaoui

**Affiliations:** 1 Laboratory of Applied Chemistry and Biomass, Faculty of Sciences Semlalia, Cadi Ayyad University, Bd Prince Moulay Abdellah, Marrakech 40000, Morocco; elmehdi.darrag@ced.uca.ma (E.M.D.); romane@uca.ac.ma (A.R.); bacaoui@uca.ac.ma (A.B.); 2 Abiotic Stress Physiology Team, CNRST-Labeled Research Unit (URL05-CNRST), AgroBiotech Center, Laboratory of Excellence in Agrobiotechnology and Bioengineering, Faculty of Sciences Semlalia, Cadi Ayyad University, Marrakech 40000, Morocco; a.meddich@uca.ac.ma; 3 Water Sciences, Microbial Biotechnologies and Sustainability of Natural Resources Laboratory (Aquabiotech), Faculty of Sciences Semlalia, Cadi Ayyad University, Av. Prince My Abdellah, P.O. Box 2390, Marrakech 40000, Morocco; yasser.essadki@ced.uca.ma (Y.E.); oudra@uca.ac.ma (B.O.); 4 Molecular Chemistry Laboratory, Faculty of Sciences Semlalia, Cadi Ayyad University, Bd. Prince My Abdellah, P.O. Box 2390, Marrakech 40000, Morocco; s.zekri.ced@uca.ac.ma (S.Z.); i.hdoufane@uca.ac.ma (I.H.); cherqaoui@uca.ma (D.C.); 5 Laboratory of Physical Chemistry of Materials and Environment, Department of Chemistry, Faculty of Sciences Semlalia, Cadi Ayyad University, P.O. Box 2390, Marrakech 40000, Morocco; h.chernane@uca.ac.ma; 6 Sustainable Materials Research Center (SUSMAT-RC), University of Mohammed VI Polytechnic, Benguerir 43150, Morocco; 7 Interdisciplinary Centre of Marine and Environmental Research (CIIMAR), Terminal de Cruzeiros do Porto de Leixões, Av. General Norton de Matos, s/n, 4450-208 Porto, Portugal; 8 Department of Biology, Faculty of Sciences, University of Porto, Rua do Campo Alegre, 4169-007 Porto, Portugal

**Keywords:** harmful algal blooms, monoterpenes, *Microcystis aeruginosa*, *Chlorella sorokiniana*, molecular docking, algaecide selectivity, ADMET

## Abstract

Harmful algal blooms pose a persistent threat to the integrity of freshwater ecosystems and public health. However, there are no selective chemical control agents available to suppress cyanobacterial growth without damaging beneficial phytoplankton. In this study, ten structurally diverse monoterpenes were assessed in vitro for their differential activity against the potent toxin-producing cyanobacterium *Microcystis aeruginosa* and the ecologically valuable microalga *Chlorella sorokiniana* using disc diffusion (DDM) and minimum inhibitory concentration (MIC) assays. Inhibition zones against *M. aeruginosa* ranged from 6.9 to 43.6 mm, with thymol recording the largest zone (43.6 mm). MIC values ranged from 0.25 to >1 mg/mL for both organisms, and selectivity indices identified camphor and carvone as the most cyanobacterium-preferential compounds, while carene and α-pinene showed the inverse selectivity pattern. Molecular docking against six AlphaFold2-predicted target proteins, photosynthetic complexes, Adenosine Triphosphate (ATP) synthase subunits, and superoxide dismutase (SOD) from both organisms, revealed binding affinities between −3.9 and −6.2 kcal/mol. Phenolic monoterpenes consistently engaged active-site glutamate and aspartate residues via hydrogen bonds and Pi–Anion interactions, most strikingly in the *M. aeruginosa* ATP synthase, whereas the *M. aeruginosa* SOD represented the least amenable target for all compounds. Computational ADMET profiling confirmed favorable pharmacokinetic properties and low predicted toxicity for the full panel.

## 1. Introduction

Cyanobacteria such as *Microcystis aeruginosa* and microalgae like *Chlorella sorokiniana* are often coexisting in the same aquatic environments as common components of freshwater ecosystems with significantly different ecological roles and impacts. Driven by eutrophication, high temperatures, and nutrient enrichment, *M. aeruginosa* is a bloom-forming cyanobacterium that produces microcystins that pose documented threats to aquatic biodiversity, water quality, and public health [1]. Its blooms are particularly high in nitrogen and phosphorus, leading to harmful algal blooms (HABs), which cause oxygen depletion and disrupt aquatic food webs [2]. In contrast, *C. sorokiniana* is a eukaryotic microalga with recognized environmental benefits, including carbon sequestration, wastewater treatment, and biotechnological applications across food, pharmaceutical, and wastewater treatment industries, underscoring the importance of preserving its viability when evaluating candidate algicidal agents [3,4,5,6].

In freshwater systems, *M. aeruginosa* and *C. sorokiniana* often compete for light, nutrients, and space, where they coexist within mixed phytoplankton communities [7]. Under high nutrient loads, reduced grazing pressure, and elevated temperatures, studies have shown that cyanobacteria such as *Microcystis* outcompete microalgae, leading to cyanobacterial dominance in eutrophic waters [8,9]. To manage these blooms, current approaches include physical removal, nutrient load reduction, and chemical algaecides, such as copper sulfate and synthetic herbicides [10,11], yet physical interventions are often cost-prohibitive for large-scale applications, and chemical algaecides lack species specificity, causing collateral damage to non-target photosynthetic organisms, including beneficial green algae that contribute to ecosystem stability and water quality [12]. Additionally, the application of broad-spectrum algaecides triggers the release of intracellular toxins from lysed cyanobacterial cells, exacerbating water contamination [13,14], which justifies an urgent need for selective biocontrol agents to suppress cyanobacterial growth while minimizing adverse effects on desirable phytoplankton communities.

Monoterpenes are a diverse group of volatile secondary metabolites that have gained increasing attention as potential antimicrobial agents because of their broad-spectrum bioactivity and natural origin [15,16]. Studies on these C_10_ isoprene derivatives show that they possess antioxidant, antimicrobial, antifungal, and herbicidal effects, with their mechanisms of action such as membrane disruption, enzyme inhibition, and interference with cellular energy processes [17], and recent research demonstrates the algicidal activity of specific monoterpenes against cyanobacteria [18]. Most monoterpenes are environmentally degradable and are quickly broken down by microorganisms, which makes them promising candidates for managing HABs.

While in vitro assessments provide essential data on antimicrobial efficacy, they offer limited insight into the pharmacokinetic and toxicological profiles required to assess the practical applicability and environmental safety of candidate compounds; thus, an in silico prediction of absorption, distribution, metabolism, excretion, and toxicity (ADMET) parameters has emerged as an informative complementary approach in drug discovery and environmental chemical assessment [19,20]. Integrating computational ADMET profiling with experimental bioactivity data can further evaluate the translational potential of bioactive natural products, identifying compounds that exhibit not only desirable antimicrobial properties but also favorable safety profiles [21]. Additionally, molecular docking studies provide valuable mechanistic insights by predicting the binding affinities and interaction patterns of bioactive compounds with target proteins, thereby elucidating potential modes of action at the molecular level [22].

The present study was designed to characterize the in vitro activity of a panel of ten monoterpenes against *M. aeruginosa* and *C. sorokiniana* and quantify their selectivity using MIC-based indices, thereby elucidating structure–activity and structure–selectivity relationships. Additionally, this study integrates molecular docking on key photosynthetic and stress-related targets with ADMET predictions to link chemical features to both target engagement and predicted environmental/toxicological behavior, an approach rarely implemented in the context of natural algaecides.

## 2. Results

### 2.1. Anti-Cyanobacterial and Anti-Microalgal Potential

The selected monoterpenes were evaluated for their anti-cyanobacterial and anti-microalgal potential qualitatively, using the disc diffusion method. Compared with copper sulfate at 30 mg/mL as a standard antibacterial agent, DMSO was used as a negative control, which showed no effect in the experiments. The results (Table 1) revealed that five of the tested compounds showed activity against *M. aeruginosa* with inhibiting diameters ranging from 6.9 mm to 43.5 mm, with thymol showing the highest activity. As for *C. sorokiniana*, nine out of the 10 tested monoterpenes showed activity. The inhibition diameters ranged from 8.6 mm to 29.2 mm, with carvacrol showing the highest inhibition.

**Figure 1:**
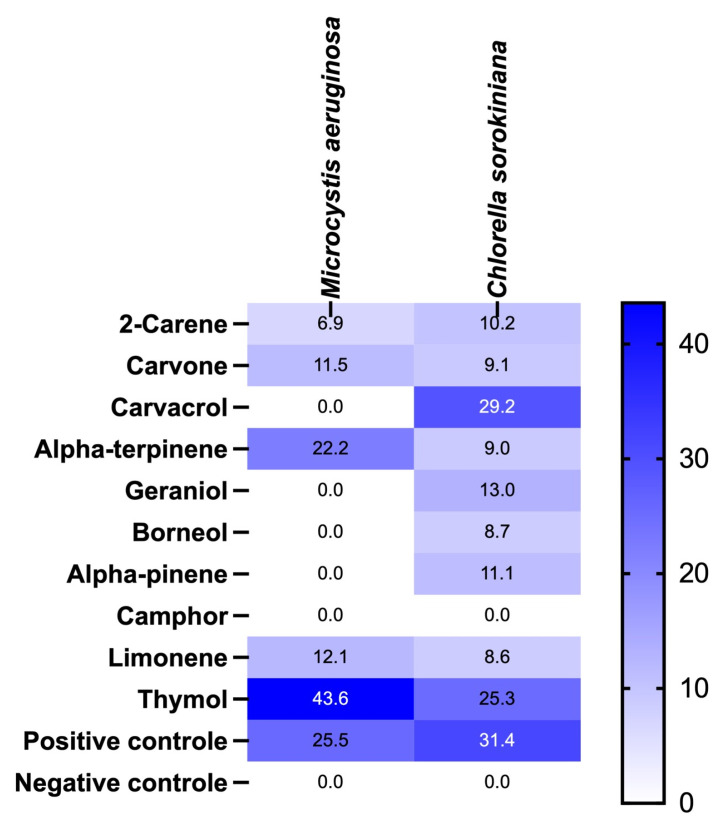
Comparative heatmap of monoterpenoid inhibition diameters against *M. aeruginosa* and *C. sorokiniana* (color intensity is proportional to DDM means).

The heatmap results (Figure 1) reveal an interesting selectivity pattern among the tested monoterpenoids against *M. aeruginosa* and *C. sorokiniana*. Some compounds demonstrated a pronounced anti-cyanobacterial activity while having a minimal inhibitory effect on *C. sorokiniana*, suggesting potential use as selective inhibitors of harmful cyanobacteria without significantly affecting beneficial or neutral green algae.

Among the tested compounds, alpha-terpinene and limonene exhibited relatively strong inhibitory effects on *Microcystis* (22.2 mm and 12.1 mm inhibition zones, respectively) while causing only mild to moderate inhibition in *Chlorella* (9 mm and 8.6 mm, respectively). Similarly, 2-carene and carvone moderately inhibited both organisms but showed slightly higher activity on *M. aeruginosa* than on *C. sorokiniana*, indicating partial selectivity. Thymol, in contrast, showed a very strong inhibitory effect against *M. aeruginosa* (43.57 mm inhibition zone) and a substantial, but less pronounced, activity on *C. sorokiniana* (25.27 mm). This indicates that thymol is a non-highly selective inhibitor but extremely effective against cyanobacteria.

On the other hand, minimum inhibitory concentration (MIC) assays revealed an appreciable variability in the activity of the tested monoterpenes against *M. aeruginosa* and *C. sorokiniana*.

The lowest MIC values for *M. aeruginosa* (0.25 mg/mL) were recorded for carvacrol, carvone, and thymol, followed by camphor, 2-carene, and alpha-pinene (0.5 mg/mL), whereas borneol exhibited a higher MIC of 1 mg/mL, and alpha-terpinene, geraniol, and limonene did not reach complete growth inhibition at the highest concentration tested (>1 mg/mL). As for *C. sorokiniana*, the most potent monoterpenes were 2-carene, alpha-pinene, and thymol, with MIC values of 0.125 mg/mL, followed by carvacrol and carvone, at 0.25 and 0.5 mg/mL, respectively, while borneol, alpha-terpinene, geraniol, and limonene inhibited growth only at 1 mg/mL, and camphor remained inactive at the tested concentrations.

The comparison between the disk diffusion method and MIC results generally showed good qualitative agreement, which confirms that the most active compounds identified by DDM are genuinely potent in a more qualitative format. However, some discrepancies were observed, mostly for carvacrol against *M. aeruginosa*, where it showed a low MIC of 0.25 mg/mL but no inhibition zone. Such divergence is well documented for hydrophobic and volatile essential oil constituents, as their poor diffusion through agar, partial loss by volatilization, and adsorption to the matrix can lead to the underestimation of their activity in DDM despite their clear inhibition in microdilution tests. Therefore, the MIC values should be considered more reliable for evaluating the potency of these monoterpenes in this experiment.

**Figure 2:**
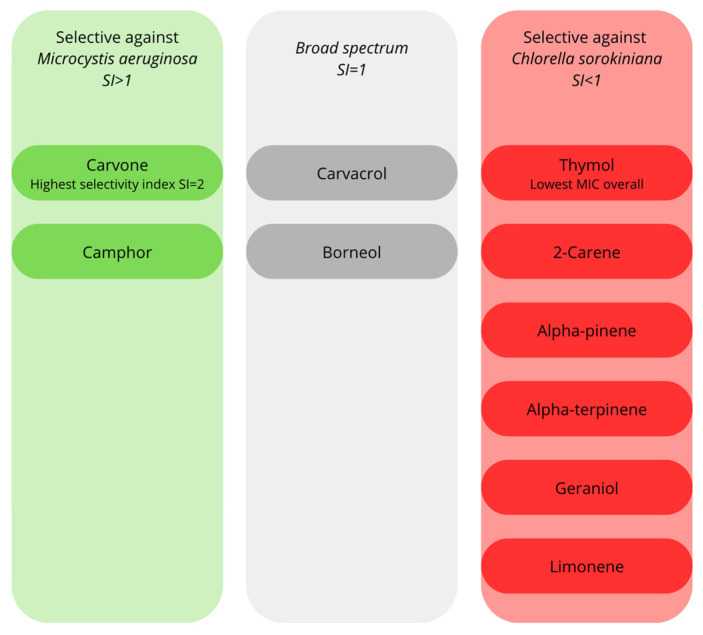
Summary of the differential antialgal activity of the tested monoterpenes. Compounds are organized by selectivity index (SI = MIC *C. sorokiniana*/MIC *M. aeruginosa*) into three zones: preferential activity against *M. aeruginosa* (SI > 1, green), broad-spectrum activity (SI = 1, gray), and preferential activity against *C. sorokiniana* (SI < 1, red).

Carvacrol and borneol showed limited selectivity, with SI values around 1.0, indicating similar susceptibility of both targets when carene, limonene, geraniol, α-terpinene, and α-pinene displayed SI values lower than 1, reflecting a stronger inhibitory effect on *C. sorokiniana* than on *M. aeruginosa*. As for thymol, with a selectivity index lower than 1, it remained slightly more active against the green alga. In contrast, camphor and carvone exhibited a modest preference for inhibiting *M. aeruginosa* relative to *C. sorokiniana* under the present experimental conditions. Among all tested compounds, camphor and carvone showed the most promising results in terms of selectivity against the tested cyanobacterial strain (Figure 2); nevertheless, the other monoterpenes could be considered as broadly non-selective algicides.

### 2.2. ADMET (Absorption, Distribution, Metabolism, Excretion, Toxicity)

The ADME (absorption, distribution, metabolism, excretion) prediction showed that all ten monoterpenes complied with Lipinski, Egan, and Veber rules (Table 2), with no Lipinski violations, which indicates generally a favorable drug-likeness. All compounds exhibited moderate lipophilicity, with consensus LogP values between 2.37 (camphor) and 3.44 (α-pinene), and low polar surface areas (TPSA ≤ 20.23 Å^2^), indicating a good membrane permeability. Gastrointestinal absorption was predicted to be high for most molecules, except for α-terpinene, limonene, 2-carene, and α-pinene, while every compound showed a bioavailability score of 0.55 and was predicted to cross the blood–brain barrier. None of the monoterpenes was identified as a P-gp substrate, and only thymol and carvacrol were predicted as CYP1A2 inhibitors, whereas limonene and α-pinene showed potential CYP2C9 inhibition. The skin permeability (Log Kp) values were in the range of approximately −5.7 to −3.9 cm/s, suggesting limited dermal penetration, and no PAINS alerts were detected for any compound. Brenk structural alerts were absent for borneol, thymol, carvacrol, and camphor, whereas the more unsaturated terpenes (α-terpinene, limonene, 2-carene, carvone, geraniol, and α-pinene) all carried a single isolated alkene alert.

The ProTox results (Table 3) indicated that all ten monoterpenes did not show any toxicity in all tested parameters, which were classified as non-hepatotoxic (NH), with probability scores for absence of hepatotoxicity ranging from 0.65 (carvone) to 0.86 (α-pinene). Carcinogenicity models categorized every compound as non-carcinogenic (NC), with probabilities between 0.60 and 0.83, while immunotoxicity and mutagenicity were uniformly predicted as negative, with high confidence values across the panel. The absence of cytotoxicity suggests a relatively low risk of nonspecific mammalian cytotoxicity at systemic levels, and the predicted oral LD50 values in rodents ranged from 500 to 4800 mg/kg, placing thymol, carvacrol, borneol, camphor, α-terpinene, and carvone in toxicity class 4, whereas 2-carene, limonene, α-pinene, and geraniol fell into the less toxic class 5.

The in silico ecotoxicological assessment of the ten selected compounds was carried out using validated QSAR models implemented in the VEGA HUB platform, covering bioaccumulation, acute aquatic toxicity, chronic endpoints, and terrestrial toxicity (Table 4). Overall, the results revealed a moderate-to-low environmental hazard profile for most compounds, with notable differences depending on the structural features of each molecule. Regarding bioaccumulation, bioconcentration factor (BCF) values predicted by the Arnot–Gobas model ranged from 1.52 to 3.34 log(L/kg). Molecules 2-carene, limonene, alpha-pinene, and alpha-terpinene exhibited BCF values approaching or exceeding 3.0 log(L/kg), which is close to the regulatory threshold of 3.3 log(L/kg) established under REACH legislation for bioaccumulative substances, suggesting that these compounds warrant further experimental evaluation. The remaining molecules displayed BCF values below 2.5 log(L/kg), indicating a lower tendency for bioaccumulation. With respect to acute fish toxicity, the Fathead Minnow LC50 values (EPA model) varied considerably across the series, ranging from 1.84 mg/L (2-carene) to 100 mg/L (borneol). According to the GHS classification criteria, compounds with LC50 below 1 mg/L are considered acutely toxic to fish (Category 1), while values between 1 and 10 mg/L fall under Category 2. 2-carene and limonene exhibited the lowest LC50 values (1.84 and 5.15 mg/L, respectively), suggesting a relatively higher acute fish toxicity compared to the other derivatives. In contrast, borneol and camphor showed LC50 values of 100 and 67.93 mg/L, respectively, placing them in a lower aquatic hazard category. For Daphnia magna, EC50 values predicted by the IRFMN model ranged from 1.10 mg/L (Limonene) to 21.96 mg/L (carvone), with the majority of compounds exhibiting EC50 values between 1 and 10 mg/L, consistent with moderate acute toxicity to crustaceans. Algae EC50 values, estimated using the ProtoQSAR-Combase model, were generally higher, ranging from 2.24 to 74.65 mg/L, suggesting that algae are comparatively less sensitive to these compounds than fish or Daphnia magna. Regarding chronic toxicity, the predicted NOEC values for Daphnia magna and algae (IRFMN models) were systematically lower than the corresponding acute EC50 values, which is consistent with the expected acute-to-chronic ratio for environmental hazard assessment. Fish chronic NOEC predictions were interpreted with caution, as most compounds fell outside the applicability domain of the model. Earthworm NOEC values (CONCERT model) ranged from 11.13 mg/Kg (carvone) to 197.07 mg/Kg (geraniol), suggesting limited terrestrial ecotoxicity risk.

### 2.3. Binding Pockets Prediction

**Figure 3:**
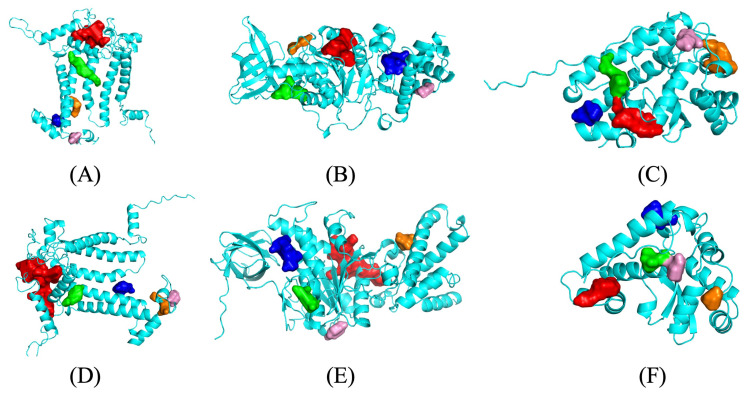
Binding pockets of the target proteins: (**A**) photosystem II D1 protein (psbA1) (UniProt ID: W8SIR2); (**B**) ATP synthase subunit beta (UniProt ID: W8TIQ2); and (**C**) superoxide dismutase (UniProt ID: A0A2P6U0Q9) from *C. sorokiniana*, as well as (**D**) photosystem II D1 protein (PsbA) (UniProt ID: P51764); (**E**) ATP synthase subunit beta (UniProt ID: B0JFM7); and (**F**) superoxide dismutase [Cu-Zn] (UniProt ID: A0A857DAR8) from *M. aeruginosa*.

Binding pockets were identified for all six AlphaFold2-predicted target models using POCASA and validated with CB-Dock, which defined the grid search spaces subsequently used for docking. The top-ranked primary cavities differed substantially across functional classes (Table 5). The top five cavities are presented in Figure 3. The photosynthetic proteins presented the largest sites. AF-P51764-F1 (*M. aeruginosa*) returned a primary pocket of 812 Å^3^ (VD = 2527), the most spacious cavity identified across the full target panel, while AF-W8SIR2-F1 (*C. sorokiniana*) yielded a primary pocket of 398 Å^3^ (VD = 1252). ATP synthase subunits occupied an intermediate range, with near-equivalent cavities for both organisms: 394 Å^3^ (VD = 987) for AF-B0JFM7-F1 and 379 Å^3^ (VD = 1000) for AF-W8TIQ2-F1. The superoxide dismutase models returned markedly restricted primary sites: 80 Å^3^ (VD = 212) for AF-A0A857DAR8-F1 and 94 Å^3^ (VD = 266) for AF-A0A2P6U0Q9-F1.

### 2.4. Molecular Docking

Molecular docking of the ten monoterpenes against three paired targets from *M. aeruginosa* and *C. sorokiniana* photosynthetic complexes (PsbA/AF-P51764-F1 and AF-W8SIR2-F1), ATP synthase subunits (AF-B0JFM7-F1 and AF-W8TIQ2-F1), and superoxide dismutase (AF-A0A857DAR8-F1 and AF-A0A2P6U0Q9-F1) yielded binding free energies spanning −3.9 to −6.2 kcal/mol across the full compound-target matrix (Table 6). Scores differed substantially both among compounds and among targets, indicating that binding is governed by structural complementarity rather than non-selective hydrophobic partitioning.

The *M. aeruginosa* ATP synthase was the most receptive target overall. Alpha-terpinene and carvacrol each recorded the highest affinity observed in the study (−6.2 kcal/mol), followed by carvone (−6.0 kcal/mol) and limonene (−6.1 kcal/mol). Against the *C. sorokiniana* photosynthetic protein, carvone and carvacrol also achieved strong binding (−6.1 and −6.0 kcal/mol, respectively), while thymol led against the *C. sorokiniana* ATP synthase with −5.9 kcal/mol. In sharp contrast, the *M. aeruginosa* SOD was the least responsive receptor across all ten compounds, with affinities confined to a narrow band of −3.9 to −4.4 kcal/mol, suggesting a binding site poorly suited to the monoterpenoid scaffold.

**Figure 4:**
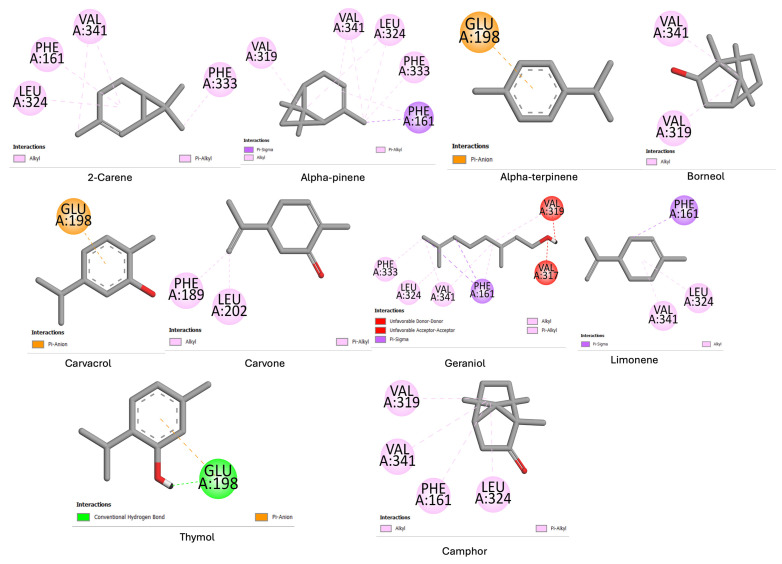
2D scheme of the protein–ligand interactions between the selected monoterpenes and the ATP synthase (W8TIQ2) of *Chlorella sorokiniana*.

**Figure 5:**
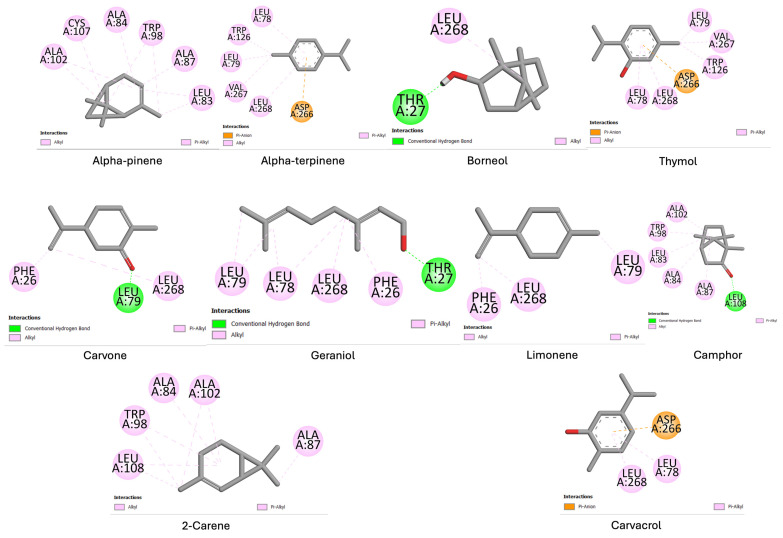
2D scheme of the protein–ligand interactions between the selected monoterpenes and the SOD (A0A2P6U0Q9) of *Chlorella sorokiniana*.

**Figure 6:**
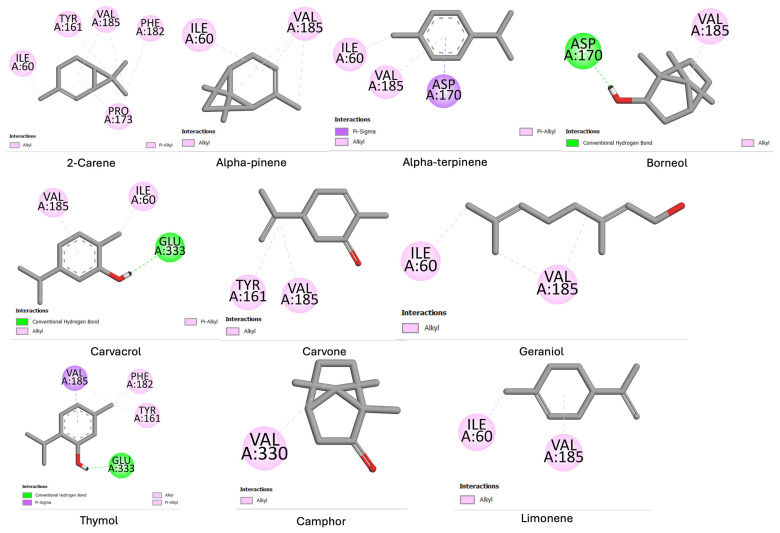
2D scheme of the protein–ligand interactions between the selected monoterpenes and the photosynthesis system (W8SIR2) of *Chlorella sorokiniana*.

**Figure 7:**
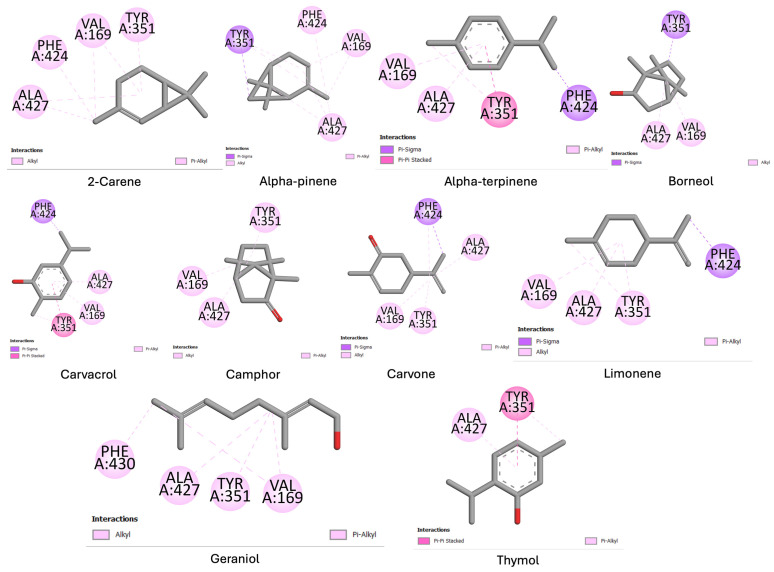
2D scheme of the protein–ligand interactions between the selected monoterpenes and the ATP synthase (B0JFM7) of *Microcystis aeruginosa*.

**Figure 8:**
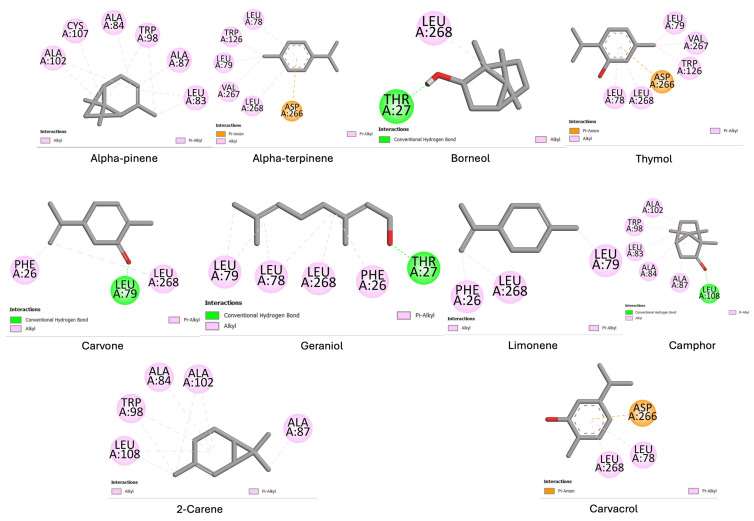
2D scheme of the protein–ligand interactions between the selected monoterpenes and the SOD (A0A857DAR8) of *Microcystis aeruginosa*.

**Figure 9:**
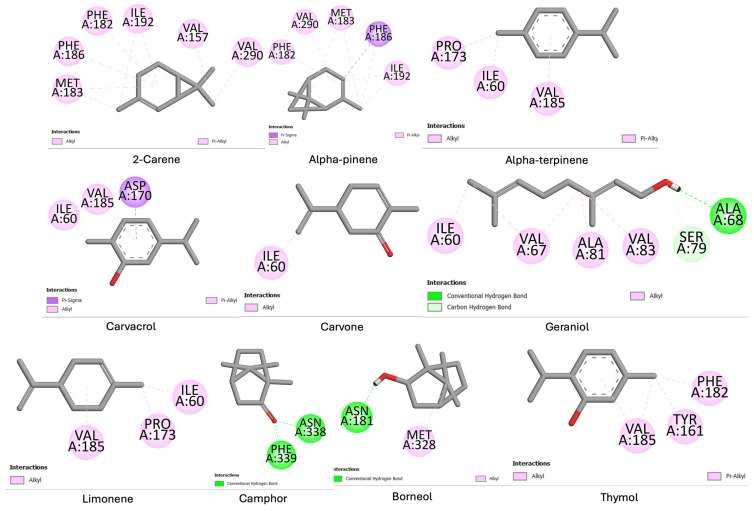
2D scheme of the protein–ligand interactions between the selected monoterpenes and the photosynthesis (P51764) of *Microcystis aeruginosa*.

Analysis of binding interaction profiles revealed clear mechanistic differences between phenolic and non-phenolic monoterpenes (Figure 4, Figure 5, Figure 6, Figure 7, Figure 8 and Figure 9) (Appendix A). At the *C. sorokiniana* ATP synthase, thymol established a conventional hydrogen bond with GLU198 (2.38 Å) and a simultaneous Pi–Anion electrostatic interaction with the same residue, a dual-mode contact absent from all non-phenolic compounds. Carvacrol formed an analogous Pi–Anion contact at GLU198 (4.71 Å) but without the direct H-bond, consistent with its slightly lower score at this target. At the *M. aeruginosa* photosynthetic protein PsbA, carvacrol and thymol recorded the highest affinities (−5.9 and −5.8 kcal/mol) through comparable electrostatic engagement of active-site residues. The purely hydrocarbon monoterpenes alpha-pinene, limonene, 2-carene, and camphor relied throughout on networks of alkyl and Pi–Alkyl contacts with hydrophobic residues (VAL, LEU, ALA, PHE), forming interaction profiles that were extensive in contact number but lower in energy than those of the phenolics. A noteworthy exception was α-terpinene, whose fully conjugated diene system engaged ASP266 of the *M. aeruginosa* ATP synthase through a Pi–Anion interaction (3.34 Å), accounting for its joint top score at that target despite carrying no polar functional group. Borneol and camphor established conventional H-bonds at several targets, camphor to ASN338 and PHE339 of the *C. sorokiniana* SOD (2.23 and 2.12 Å), and borneol to THR27 of the *C. sorokiniana* SOD (1.82 Å) and ASP170 of W8SIR2 (2.52 Å), yet their overall affinity scores remained among the lowest in the panel, suggesting that H-bond count alone is insufficient when the surrounding hydrophobic complementarity is limited.

## 3. Discussion

The antimicrobial activities observed in the present study align with and extend previous investigations into the algaecidal properties of monoterpenes [23,24,25,26], while revealing novel insights into their selectivity profiles. In research conducted by Balcerzak et al. [27], several monoterpenoids were tested for their ability to inhibit the growth of various cyanobacterial species. Notably, (+)-3-carene emerged as the most effective inhibitor, significantly reducing the growth of *Chroococcus minutus* and *Nodularia moravica*, leading to very low chlorophyll levels and even death of N. moravica after 14 days of culture. The study highlighted that the inhibitory effects of monoterpenoids can vary across cyanobacterial consortia. For instance, the addition of monoterpenoids limited the development of a mixed culture of *Anabaena* sp., *Chroococcus minutus*, and *Nodularia moravica*, indicating that species interactions can influence the overall response to these compounds. This research also suggested that cyanobacteria may activate cellular defense mechanisms in response to monoterpenoids, transforming more toxic compounds into less harmful forms. This detoxification process was observed with the transformation of (+)-carvone into (+)-dihydrocarvone by the cyanobacteria. The study also indicates that the effectiveness of monoterpenoids in inhibiting cyanobacterial growth is dependent on both the type of monoterpenoid and the specific cyanobacterial species involved, suggesting a complex interaction that warrants further investigation.

Another study on the inhibitory effect of eugenol against *M. aeruginosa* reported that this compound significantly inhibited the growth of the cyanobacteria. The specific growth rate also decreased significantly with increasing eugenol concentrations, indicating a dose-dependent effect. The study observed a marked reduction in cell density. Eugenol exposure led to notable physiological changes in *M. aeruginosa*, including damage to cellular ultrastructure as observed through transmission electron microscopy. Additionally, eugenol exposure resulted in increased release of extracellular organic matter, including substances like soluble microbial products and humic acid-like materials [25].

Supporting the established literature, phenolic monoterpenes, particularly carvacrol and thymol, demonstrated potent antimicrobial activity against both Gram-positive and Gram-negative bacteria [28] while also exhibiting broad-spectrum antifungal activity against eukaryotic pathogens [29]. This antimicrobial spectrum encompasses both planktonic and sessile growth forms across bacterial and Candida species [30]. The experimental findings corroborate the established understanding that phenolic monoterpenes exhibit superior antimicrobial efficacy compared to their non-phenolic counterparts, attributable to the hydroxyl group’s capacity in reactive oxygen species (ROS) generation and membrane destabilization [31]. The phenolic hydroxyl group of monoterpenes, such as thymol and carvacrol, can participate in one-electron oxidation under aerobic conditions, generating phenoxyl radicals that feed into this ROS cascade, thereby amplifying intracellular oxidative stress beyond endogenous levels [32]. This exogenous ROS burden is particularly damaging in photosynthetic microorganisms, such as *C. sorokiniana* and *M. aeruginosa*, whose photosynthetic electron transport chains are themselves constitutive endogenous sources of superoxide and singlet oxygen. Hence, the monoterpene-induced ROS acts synergistically with photosynthesis-derived oxidative stress to overwhelm the cell’s antioxidant defenses. At the membrane level, biomembranes are highly susceptible to attack by biologically generated ROS, and the presence of oxidized lipids could change membrane properties, especially permeability. Molecular dynamics simulations have demonstrated that hydroxyl (HO^•^) and hydroperoxyl (HO_2_^•^) radicals can penetrate deep into the lipid headgroups region and, due to membrane fluidity and disorder, these radicals have access to potential peroxidation sites along the lipid hydrocarbon chains without having to overcome the permeation free energy barrier [33]. This is of direct relevance to the observed activity of phenolic monoterpenes: once the hydroxyl group-mediated ROS cascade generates HO^•^ and HO_2_^•^ radicals in proximity to the algal cell membrane, these radicals gain spontaneous access to the acyl chain unsaturations of membrane phospholipids and initiate lipid peroxidation. HO_2_^•^ radicals in particular were found to be an order of magnitude more concentrated in the headgroups region than in bulk water, substantially increasing the probability of their encounter with the primary initiation sites of lipid peroxidation chain reactions [34,35]. The consequences of this peroxidation result in alterations of the membrane structure, affecting its fluidity and damaging its integrity. In the context of microalgal cells, the generation of such secondary lipid peroxidation products at the thylakoid membrane could directly impair photosystem II structural integrity, the photosynthetic electron transport chain, and ultimately carbon fixation capacity, inducing a multi-target mechanism of inhibition that is mechanistically inaccessible to non-phenolic monoterpenes lacking the redox-active hydroxyl group.

However, a striking and previously unreported observation emerged in the differential selectivity patterns of these structural isomers; while carvacrol exhibited equipotent, non-selective activity (SI = 1.0), thymol displayed a two-fold greater toxicity toward *C. sorokiniana* (SI = 0.5). This differential selectivity is particularly noteworthy given that carvacrol and thymol are positional isomers, differing only in the placement of the hydroxyl group on the aromatic ring. This subtle structural variation appears to profoundly influence organism-specific toxicity, potentially due to differences in membrane partitioning coefficients, hydrogen bonding interactions with target biomolecules, or differential susceptibility to detoxification mechanisms between prokaryotic cyanobacteria and eukaryotic green algae. Previous studies have demonstrated that the position of hydroxyl groups significantly affects the hydrophobicity and membrane-penetrating ability of phenolic compounds [36], which may explain why the more lipophilic thymol exhibits enhanced activity against the sterol-containing membranes of *Chlorella* compared to the hopanoid-based membranes of *Microcystis* [37]. As both algal strains used in this study were non-axenic, the potential influence of associated heterotrophic bacteria on the observed inhibitory activity cannot be entirely excluded. Future studies using axenic strains or including bacterial enumeration alongside algal bioassays would allow a more definitive attribution of the algicidal effects to the tested monoterpenes. This study represents the first systematic comparison of monoterpene selectivity between toxic cyanobacteria and beneficial green algae, addressing a gap in the literature where previous investigations have focused predominantly on broad-spectrum antimicrobial activity without evaluating ecological selectivity, a parameter essential for developing environmentally responsible bloom management strategies [12,38]. Furthermore, the identification of camphor and carvone as a selectively cyanobactericidal agent (SI > 2.0) represents a novel finding with significant implications for targeted HAB control, as these compounds have received limited attention in phycological research despite their widespread availability and established safety profile in various applications [39,40].

The computational ADMET assessment reveals encouraging safety characteristics for the investigated monoterpenes, positioning them as viable candidates for sustainable algae management applications. The absence of predicted hepatotoxicity, carcinogenicity, immunotoxicity, and mutagenicity, and the high oral LD50 values and classifications align with the established literature describing numerous food-grade monoterpenes as generally recognized as relatively safe when administered at appropriate concentrations [41]. This safety profile is consistent with their proposed mechanism of action involving reversible membrane perturbation rather than permanent cellular damage [42].

The favorable physicochemical properties (LogP 2.3–3.4, low TPSA) support effective membrane penetration, which correlates with documented membrane-disrupting mechanisms observed in antimicrobial applications [43]. And the identification of thymol, carvacrol, camphor, and carvone as optimal lead candidates reflects their balance between demonstrated anti-cyanobacterial efficacy and acceptable safety margins. This positions them favorably for development as precision algaecides that minimize ecological disruption while maintaining effective biological activity. However, validation through comprehensive in vivo toxicity studies and ecotoxicological evaluations remains essential to confirm the promising in silico predictions and support their practical application in aquatic environments.

The ecotoxicological and ADMET profiles reported here were generated using complementary in silico platforms (VEGA HUB for aquatic and terrestrial hazard endpoints, SwissADME for pharmacokinetic and drug-likeness profiling, and ProTox-3.0 for systemic toxicity prediction), each of which carries inherent limitations that must be considered when interpreting the results in the context of environmental algicide development. SwissADME and ProTox-3.0 were developed and benchmarked predominantly against curated datasets of synthetic pharmaceutical compounds, with training sets heavily weighted toward drug-like molecules optimized for oral bioavailability and human metabolic processing. A further limitation concerns the inability of current QSAR-based ADMET models to account for environmental fate processes intrinsic to volatile terpenoids in aquatic systems, including rapid surface evaporation, UV-driven photodegradation, and microbial biotransformation, all of which would substantially reduce actual environmental exposure concentrations relative to the modelled LC_50_ and EC_50_ values, suggesting that the predicted hazard may systematically overestimate real-world risk for this compound class. Notwithstanding these constraints, the convergence of VEGA HUB predictions with the bioassay-derived MIC and selectivity index data (with borneol and camphor consistently emerging as low-hazard), cyanobacterium-preferential candidates across both experimental and computational analyses, lends confidence to the overall hazard ranking reported in this study. Empirical validation through standardized OECD guideline studies (TG 201 for algae, TG 202 for Daphnia magna, and TG 203 for fish) remains an essential next step before any of these candidates can be responsibly advanced toward field-scale bloom management applications.

From a practical and environmental engineering perspective, translating the selective in vitro efficacy of these monoterpenes into real-world harmful algal bloom (HAB) management requires overcoming challenges associated with their high volatility and low water solubility. Because raw monoterpenes are hydrophobic, their direct application to open aquatic environments risks rapid phase separation and atmospheric volatilization, which drastically reduces their bioavailable concentration in the water column. To resolve this solubility bottleneck, future field-scale applications must utilize green, biodegradable formulation and delivery systems. Incorporating bioactive monoterpenes into nano-encapsulation matrices, such as chitosan-based nanoparticles [44], lipid nano-emulsions [45], or cyclic oligosaccharide inclusion complexes, like β-cyclodextrin [46], can significantly enhance their aqueous dispersibility and thermodynamic stability. These nano-delivery architectures prevent premature volatilization, facilitate homogeneous diffusion through the water column, and enable the sustained, controlled release of the active compounds. Consequently, encapsulation ensures that optimal lethal concentrations are delivered directly to target cyanobacterial cells at a fraction of the raw material dosage, minimizing the environmental footprint while maintaining chemical stability.

The pocket volume hierarchy provides a structural rationale for the docking affinity gradient reported below. The narrow substrate channel of manganese superoxide dismutases, architecturally optimized for superoxide anion access to the catalytic metal center, cannot comfortably accommodate a C_10_ monoterpenoid skeleton regardless of substituent chemistry, explaining the uniformly low binding scores (−3.9 to −4.4 kcal/mol) at both SOD receptors. The convergence of ATP synthase pocket volumes between organisms (394 versus 379 Å^3^) is notable given the prokaryote–eukaryote sequence divergence between the two subunits and likely reflects conserved steric constraints at the nucleotide-binding cleft.

None of the six proteins has an experimentally resolved three-dimensional structure in the Protein Data Bank; all models are AlphaFold2-predicted structures [47] retrieved from the UniProt database. This represents, to the best of our knowledge, the first binding site characterization performed on any of these targets, and the absence of experimental ligand-bound reference states precludes direct validation of the pocket geometries identified here. The congruence between the predicted pocket hierarchy and the experimental in vitro selectivity data nonetheless provides empirical coherence to the computational analysis.

The docking data provide a molecular-level explanation for several trends observed in the in vitro bioassays, though a direct quantitative correspondence between binding affinity and MIC should not be assumed, given the many steps separating a docking score from whole-organism growth inhibition. Across both organisms, phenolic monoterpenes thymol and carvacrol achieved the strongest and most varied interactions, exploiting charged residues (GLU, ASP) through hydrogen bonding and electrostatic contacts unavailable to saturated or non-phenolic terpenes. The hydroxyl group on the aromatic ring appears to be the critical determinant: it enables short, directional H-bonds, while the electron-rich phenyl ring engages anionic carboxylates via Pi–Anion forces, a dual capacity that hydrocarbon terpenes cannot replicate. This mechanistic picture suggests that their in vitro potency against both *M. aeruginosa* and *C. sorokiniana* reflects genuine multi-target engagement rather than non-specific membrane lysis alone.

The moderately high affinity of alpha-terpinene for the *M. aeruginosa* ATP synthase (−6.2 kcal/mol) despite its lack of polar groups is best explained by the geometry of its conjugated diene system, which appears spatially complementary to the ASP266 binding pocket in a way not achievable by the fully saturated or monocyclic hydrocarbon congeners.

Camphor and carvone recorded the highest selectivity indices in the bioassays, indicating preferential inhibition of *M. aeruginosa* over *C. sorokiniana*. At the docking level, both compounds displayed moderately stronger affinity for the *M. aeruginosa* ATP synthase (−4.9 and −6.0 kcal/mol, respectively) than for its *C. sorokiniana* counterpart (−4.7 and −5.5 kcal/mol), a difference that, though modest in absolute terms, may reflect differences in active-site topology between the prokaryotic and eukaryotic ATP synthase subunits. Carvone in particular achieved strong binding at both the cyanobacterial ATP synthase and the microalgae photosynthetic protein, suggesting that its selective in vitro profile is more likely a consequence of differential membrane permeability or detoxification capacity between the two organisms than of absolute target-binding preference.

The uniformly low scores against *M. aeruginosa* SOD (−3.9 to −4.4 kcal/mol) are consistent with the narrow, metal-coordinating substrate channel of cyanobacterial manganese superoxide dismutases [48,49]. Disruption of the cyanobacterial antioxidant defense system by the tested monoterpenes is, therefore, unlikely to proceed through direct SOD inhibition; amplification of intracellular oxidative stress through indirect mechanisms, such as membrane damage and electron transport chain interference, remains a plausible route that the current data cannot exclude.

Taken together, the ATP synthase subunits of *M. aeruginosa* emerge from this analysis as the highest-affinity targets for the most selective compounds (carvone, camphor), making them strong candidates for follow-up enzyme inhibition assays. The capacity of phenolic monoterpenes to engage both photosynthetic complexes and ATP synthase simultaneously suggests a multi-target mode of action that, while effective against both organisms in vitro, may be leveraged toward greater selectivity through structural optimization of the isopropylphenol scaffold.

Beyond the molecular interactions identified through docking analysis, the antialgal activity of the tested monoterpenes must be interpreted within the broader context of their well-established capacity to disrupt biological membranes. Membrane damage is considered the major mechanism by which monoterpenes and related oxygenated compounds exert their antimicrobial effects; these molecules passively diffuse into and accumulate within the lipid bilayer, disrupting hydrogen bonding and lipid packing, thereby altering the membrane’s physical properties, structure, and function. This bilayer intercalation produces measurable functional consequences: monoterpenoids and phenylpropanoids decrease membrane polarity and increase permeability in a time and concentration-dependent manner, with depolarization consistently preceding permeabilization. The superior activity of phenolic monoterpenes observed in our dataset is mechanistically explained by the role of the aromatic hydroxyl group: the hydrophobicity of phenolic monoterpenes causes their penetration into lipid membranes, creating increased permeability and leakage of ions and cellular contents, ultimately leading to cell death, and the phenolic hydroxyl group is essential for this membrane-disrupting action, as carvacrol efficiently dissolves the phospholipid bilayer by aligning between the fatty acid chains. This membrane disruption is further amplified by ROS-mediated lipid peroxidation, in which hydroxyl radicals penetrate deep into the lipid headgroup region and access peroxidation sites along the hydrocarbon chains without overcoming a permeation free energy barrier, ultimately altering membrane structure, reducing fluidity, and compromising integrity. Collectively, these mechanisms (bilayer intercalation, membrane depolarization, ion leakage, and oxidative lipid peroxidation) provide a comprehensive basis for the observed antialgal activity and the consistently lower MIC values of phenolic monoterpenes relative to non-phenolic compounds in this study [50,51,52].

To empirically delineate the relative contributions of protein-targeted inhibition, membrane disruption, and oxidative stress markers to the observed algicidal activity, future work should incorporate membrane permeability assays, chlorophyll fluorescence kinetics, and a panel of oxidative stress biomarkers for the prioritized compounds. Such an integrated mechanistic framework would also permit a more rigorous interpretation of the selectivity indices reported here and strengthen the case for monoterpene-based algicides as ecological bloom management tools.

## 4. Conclusions

The panel of ten monoterpenes examined in this study displayed differential bioactivity against the cyanobacterium *Microcystis aeruginosa* and the green microalga *Chlorella sorokiniana*, both in vitro and in silico. Disc diffusion and MIC assays confirmed the activity of carvacrol and thymol as the most potent inhibitors across both organisms, while camphor and carvone demonstrated preferential activity toward the cyanobacterial target, a distinction with potential relevance to selective bloom management applications. ADMET profiling established that all ten compounds satisfy established oral drug-likeness criteria, present no Lipinski violations, and carry predicted LD50 values in the low-to-moderate toxicity range (class 4–5), supporting their overall safety margin relative to conventional algicide treatments.

Binding pocket characterization using POCASA and CB-Dock, conducted on six AlphaFold2-predicted targets for which no experimental PDB structures currently exist, identified the photosynthetic proteins and ATP synthase subunits as geometrically accessible receptors for the monoterpenoid scaffold, while the superoxide dismutase models presented cavities too constrained for productive monoterpene binding. Molecular docking confirmed this as the *M. aeruginosa* ATP synthase (AF-B0JFM7-F1) returned the highest affinities overall (down to −6.2 kcal/mol for alpha-terpinene and carvacrol), and the two compounds with the highest in vitro selectivity (carvone and camphor) consistently scored better at the cyanobacterial ATP synthase than at its *C. sorokiniana* counterpart, offering a plausible molecular basis for their differential activity. The capacity of phenolic monoterpenes to engage charged active-site residues (GLU198, ASP266) through hydrogen bonding and Pi–Anion electrostatics, a mode of interaction unavailable to non-phenolic congeners, emerges as the key structural determinant of potency within this compound class.

These results establish, for the first time, a computational target engagement profile for monoterpenes against *M. aeruginosa* and *C. sorokiniana* using structurally predicted proteins, contributing a reference dataset for future rational design efforts aimed at improving cyanobacterial selectivity. Enzyme inhibition assays were validated, and, where structurally feasible, ligand-bound co-crystallization would be necessary to confirm the predicted binding modes. The convergence between the in vitro selectivity data, the pocket geometry, and the docking affinity patterns points to camphor and carvone as priority candidates for further development as eco-compatible, selective cyanobactericidal agents.

## 5. Materials and Methods

### 5.1. Chemicals

**Figure 10:**
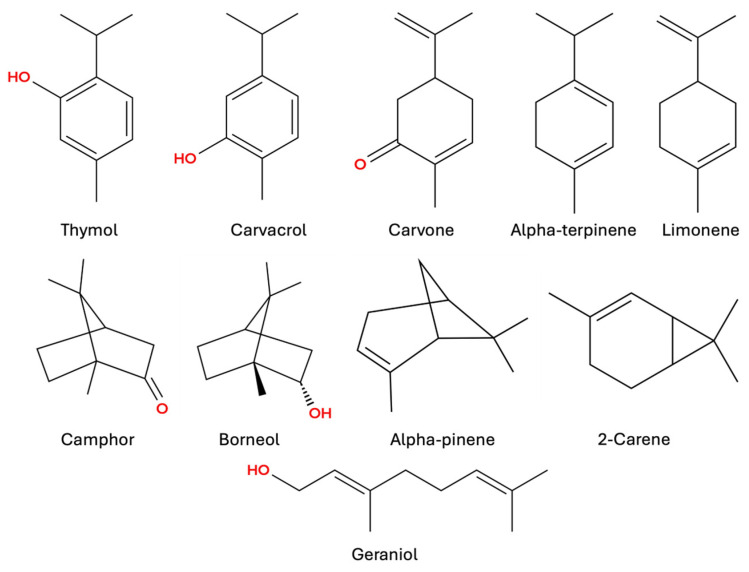
Structures of the selected monoterpenes.

Ten monoterpenes (Figure 10), borneol, alpha-terpinene, 2-carene, carvacrol, camphor, carvone, alpha-pinene, thymol, geraniol, and limonene, were obtained from Sigma Aldrich (St. Louis, MO, USA) with purity ≥ 98% and were prepared by dissolving each monoterpene in dimethyl sulfoxide (DMSO) and stored at 4 °C in amber glass vials to prevent photodegradation and volatilization.

### 5.2. Antibacterial Test

#### 5.2.1. *Microcystis aeruginosa* Strain

The strain of *M. aeruginosa* MCAUt, a non-axenic, monoclonal, and toxin-producing strain isolated from the Lalla Takerkoust eutrophic lake reservoir in 2015, was used as a model of harmful cyanobacteria [53]. A non-axenic culture was intentionally selected to preserve the natural microbiomes, thereby providing a more ecologically realistic assessment of algicidal efficacy under conditions that mimic natural bloom dynamics. After isolation and identification, the strain was maintained in culture in liquid BG11 medium under controlled conditions of light (2500 lux, photoperiod 15:9 light:dark) and temperature (25 ± 2 °C).

#### 5.2.2. *Chlorella sorokiniana* Strain

A non-axenic strain of *C. sorokiniana*, isolated in 2017 from an artificial pond in the Biology department of the Faculty of Sciences Semlalia (Marrakech), was used as a model of beneficial algae of the freshwater ecosystem [53]. Mirroring the cyanobacterial setup, the retention of the native microalgal microbiome ensures a more ecologically realistic assessment of algicidal efficacy. After isolation, the strain was maintained in culture in liquid Z8 medium under the same conditions (2500 lux, 15:9 light:dark, 25 ± 2 °C).

#### 5.2.3. Anti-Cyanobacterial and Antialgal Assay

A qualitative assessment of the antialgal/cyanobacterial potential of the studied monoterpenes in solid medium was performed during this experiment.

A modified double-layer agar plate method was used to obtain a homogeneous distribution of the microorganisms in solid medium according to the method previously described by Tazart et al. [54]. BG11 medium was used for the cyanobacterium, and Z8 medium for the microalgae.

Briefly, soft-agar medium made of 2 mL of microorganism suspension at the exponential growth phase (30 × 10^9^ cell/L (OD_600_ = 0.9 at 1 cm) for *C. sorokiniana*; 30 × 10^9^ cell/L (OD_600_ = 0.9 at 1 cm) for *M. aeruginosa* MCAUt) and 3 mL of 1% agar medium was poured onto 20 mL of basal 2% agar medium. Paper disks of 6 mm, soaked with 20 µL of a monoterpene stock solution, were prepared by dissolving each compound in pure dimethyl sulfoxide (DMSO) at a concentration of 50 mg/mL (1 mg of compound per disk); copper sulfate dissolved in sterile water at 30 mg/mL was used as a positive control (0.6 mg of copper sulfate per disk), and pure DMSO was used as a negative control, and they all were placed onto the center of the plates and immediately put at 4 °C for 2 h. To minimize evaporative loss of volatile monoterpenes during incubation, Petri dishes were sealed with Parafilm immediately after disk application and kept sealed throughout the incubation period. The double-layer agar plates were cultivated for 10 days under the previously described culture conditions. The anti-cyanobacterial and antialgal activity was expressed as mm of a clear zone around the paper disk on the double-layer agar plates. Each treatment was performed in triplicate.

#### 5.2.4. Broth Microdilution Assay

The minimum inhibitory concentration (MIC) of the ten monoterpenes, copper sulfate, and DMSO was determined using the broth microdilution method described by Zerrifi et al. [55]. Briefly, in a 96-well microplate, serial dilutions of the monoterpenes were prepared in Z8 medium supplemented with 1% DMSO, to a final volume of 100 µL per well. Then, 100 µL of a suspension of the test organisms (*M. aeruginosa* or *C. sorokiniana*) in exponential growth phase, adjusted to 4 × 10^6^ cells/mL, was added to each well, giving a final volume of 200 µL. Each plate included a solvent control (Z8 medium with 1% DMSO), a positive control (copper sulfate 30 mg/mL), and a growth control (Z8 medium with microorganism only). Plates were incubated for 8 days under the described controlled conditions of the culture chamber. The lowest concentration of the tested substance with no visible growth was recorded as the MIC. Each treatment was performed in triplicate.

#### 5.2.5. Selectivity Index

The in vitro antimicrobial assessment revealed marked variation in the efficacy and selectivity of the ten monoterpenes against *M. aeruginosa* and *C. sorokiniana*. To quantify selectivity, the selectivity index (SI) was calculated as the ratio:

SI = MIC_*Chlorella*/MIC_*Microcystis*. (1)

where SI > 1 indicates preferential activity against the cyanobacterium, SI = 1 represents non-selective activity, and SI < 1 suggests greater toxicity toward the green alga.

### 5.3. Admet Study

The pharmacokinetics (absorption, distribution, metabolism, and excretion (ADME)) studies were predicted using the ADME tool by a SwissADME online server (http://www.swissadme.ch/, accessed on 12 August 2022). The structures were exported in SMILES format, and then the drug-like and pharmacokinetic properties of the selected compounds were determined as per the developed protocol [56]. As for the toxicity, the ProTox-II webserver (http://tox.charite.de/protox_II, accessed on 12 August 2022) was used to calculate the predictions based on different parameters such as organ toxicity (hepatotoxicity), oral toxicity, and toxicological endpoints (cytotoxicity, mutagenicity, carcinotoxicity, and immunotoxicity) [57].

To complement these analyses and address the environmental hazard profile of the studied compounds, in silico ecotoxicological predictions were performed using the VEGA HUB platform (https://www.vegahub.eu/, accessed on 26 May 2026). Multiple validated QSAR models were employed to estimate aquatic toxicity endpoints across three standard organisms: fish acute toxicity (Fathead Minnow LC50, EPA model), crustacean acute toxicity (Daphnia magna EC50, IRFMN model), and algae acute toxicity (EC50, ProtoQSAR-Combase model). Chronic no-observed-effect concentrations (NOECs) were also predicted for fish, Daphnia magna, and algae using the corresponding IRFMN models. Additionally, the bioconcentration factor (BCF) was estimated using the Arnot–Gobas model, and terrestrial toxicity was assessed via the earthworm NOEC CONCERT model [58].

### 5.4. Molecular Docking

A significant challenge in conducting molecular docking studies with *M. aeruginosa* and *C. sorokiniana* is the limited availability of experimentally resolved three-dimensional protein structures for these specific organisms in the Protein Data Bank (PDB). To address this limitation and to investigate potential mechanisms underlying the observed antimicrobial selectivity of monoterpenes, key proteins involved in essential metabolic pathways common to photosynthetic microorganisms were selected: photosynthesis, cellular respiration, and oxidative stress response. Target proteins were identified through the Universal Protein Resource (UniProt) database. They included: (A) photosystem II D1 protein (psbA1) (UniProt ID: W8SIR2); (B) ATP synthase subunit beta (UniProt ID: W8TIQ2); and (C) superoxide dismutase (UniProt ID: A0A2P6U0Q9) from *C. sorokiniana*, (D) as well as photosystem II D1 protein (PsbA) (UniProt ID: P51764); (E) ATP synthase subunit beta (UniProt ID: B0JFM7); and (F) superoxide dismutase [Cu-Zn] (UniProt ID: A0A857DAR8) from *M. aeruginosa*. For the structures that did not show a co-crystallized ligand, a binding site prediction was performed using the online server POCASA [59] and CB-Dock2 [60]. POCASA identifies ligand-binding sites by rolling a probe sphere over the protein surface to detect pockets, which are subsequently ranked based on their position and size. The cavity with the highest score was selected, and the CB-Dock2 tool helped by estimating the size of the centers to guide molecular docking using AutoDock Vina [61].

The monoterpene structures were obtained from the PUBCHEM database (https://pubchem.ncbi.nlm.nih.gov/, accessed on 10 February 2025) in the SDF (structure data file) format. The selected compounds were thymol (CID: 6989), carvacrol (CID: 10364), αpinene (CID:6654), α-terpinene (CID: 7462), borneol (CID: 64685), geraniol (CID: 637566), 2-carene (CID: 79044), carvone (CID: 7439), camphor (CID: 2537), and limonene (CID: 22311). Structures of the ligands in their SDF format were then energy minimized using the MMFF94 force field. Further, the ligands were converted into AutoDock Ligand format (PDBQT) with the help of the open Babel software (version 3.1.1) [62]. To determine the binding affinity and identify the various ligand–receptor interactions responsible for the antioxidant and phytotoxic activities, molecular docking of the selected monoterpenes was performed using AutoDock Vina (version 1.2.5). The AutoDock tools software was used to validate the grid box coordinates and dimensions [63]. Biovia Discovery Studio-2021 Client and PyMOL software (version 4.6.0) were used for the visualization of 2D and 3D interactions of docking poses [64,65].

## Figures and Tables

**Table 1:** Disc diffusion method and minimal inhibitory concentrations of the selected monoterpenes (SI: selectivity index).

	DDM		MIC		
Compound	*Microcystis aeruginosa*	*Chlorella sorokiniana*	*Microcystis aeruginosa*	*Chlorella sorokiniana*	SI (*Chlorella*/*Microcystis*)
2-Carene	6.93 ± 0.72 mm	10.20 ± 0.46 mm	0.5 ± 0 mg/mL	0.125 ± 0 mg/mL	0.25
Carvone	11.53 ± 0.23 mm	9.07 ± 0.12 mm	0.25 ± 0 mg/mL	0.5 ± 0 mg/mL	2.00
Carvacrol	0.00 ± 0.00 mm	29.20 ± 0.35 mm	0.25 ± 0 mg/mL	0.25 ± 0 mg/mL	1.00
Alpha-terpinene	22.20 ± 0.21 mm	9.00 ± 0.20 mm	>1 mg/mL	1 ± 0 mg/mL	<1.00
Geraniol	0.00 ± 0.00 mm	13.03 ± 0.55 mm	>1 mg/mL	1 ± 0 mg/mL	<1.00
Bornéol	0.00 ± 0.00 mm	8.67 ± 0.57 mm	1 ± 0 mg/mL	1 ± 0 mg/mL	1.00
Alpha-pinène	0.00 ± 0.00 mm	11.13 ± 0.23 mm	0.5 ± 0 mg/mL	0.125 ± 0 mg/mL	0.25
Camphor	0.00 ± 0.00 mm	0.00 ± 0.00 mm	0.5 ± 0 mg/mL	>1 mg/mL	>1.00
Limonène	12.10 ± 0.35 mm	8.60 ± 0.17 mm	>1 mg/mL	1 ± 0 mg/mL	<1.00
Thymol	43.57 ± 0.12 mm	25.27 ± 0.06 mm	0.25 ± 0 mg/mL	0.125 ± 0 mg/mL	0.50
Negative control	0 ± 0 mm	0 ± 0 mm	>1 mg/mL	>1 mg/mL	-
Positive control	25.5 0 ± 0 mm	31.1 0 ± 0 mm	4.69 × 10^−3^ mg/mL	4.69 × 10^−3^ mg/mL	1

**Table 2:** ADME properties of the tested monoterpenes.

Property	Borneol	Thymol	Carvacrol	Camphor	Alpha-Terpinene	Limonene	2-Carene	Carvone	Geraniol	Alpha-Pinene
Molecular weight	154.25	150.22	150.22	152.23	136.23	136.23	136.23	150.22	154.25	136.23
Num. heavy atoms	11	11	11	11	10	10	10	11	11	10
Num. arom. heavy atoms	0	6	6	0	0	0	0	0	0	0
Fraction Csp3	1.00	0.40	0.40	0.90	0.60	0.60	0.80	0.50	0.60	0.80
Num. rotatable bonds	0	1	1	0	1	1	0	1	4	0
Num. H-bond acceptors	1	1	1	1	0	0	0	1	1	0
Num. H-bond donors	1	1	1	0	0	0	0	0	1	0
Molar refractivity	46.60	48.01	48.01	45.64	47.12	47.12	45.22	47.32	50.40	45,22
TPSA (Å2)	20.23	20.23	20.23	17.07	0.00	0.00	0.00	17.07	20.23	0.00
Consensus log Po/w	2.38	2.80	2.82	2.37	3.30	3.37	3.12	2.44	2.74	3.44
Lipinski rules	yes	yes	yes	yes	yes	yes	yes	yes	yes	yes
Lipinski violation	0	0	0	0	0	0	1	0	1	1
Bioavailability score	0.55	0.55	0.55	0.55	0.55	0.55	0.55	0.55	0.55	0.55
GI absorption	high	high	high	high	low	low	low	high	high	low
BBB permeant	yes	yes	yes	yes	yes	yes	yes	yes	yes	yes
P-gp substrate	no	no	no	no	no	no	no	no	no	no
CYP1A2 inhibitor	no	yes	yes	no	no	no	no	no	no	no
CYP2C19 inhibitor	no	no	no	no	no	no	no	no	no	no
CYP2C9 inhibitor	no	no	no	no	no	yes	no	no	no	yes
CYP2D6 inhibitor	no	no	no	no	no	no	no	no	no	no
CYP3A4 inhibitor	no	no	no	no	no	no	no	no	no	no
Log Kp (cm/s)	−5.31	−4.87	−4.74	−5.67	−4.11	−3.89	−5.11	−5.29	−4.71	−3.95
PAINS	0	0	0	0	0	0	0	0	0	0
Brenk	0	0	0	0	0	1 alert: isolated alkene	1 alert: isolated alkene	1 alert: isolated alkene	1 alert: isolated alkene	1 alert: isolated alkene
Synthetic accessibility	3.43	1.00	1.00	3.22	3.63	3.46	3.84	3.33	2.58	4.44
Egan rule	yes	yes	yes	yes	yes	yes	yes	yes	yes	yes
Veber rule	yes	yes	yes	yes	yes	yes	yes	yes	yes	yes

**Table 3:** Toxicity profile prediction of the tested monoterpenes (Pr: prediction, Pb: probability, NH: non-hepatotoxic, NC: non-carcinogenic, NI: non-immunotoxic, NM: non-mutagenic, NCy: non-cytotoxic, LD: lethal dose).

	Hepatotoxicity		Carcinogenicity		Immunotoxicity		Mutagnicity		Cytotoxicity		Predicted LD50 (mg/kg)	Toxicity Class
Compounds	Pr	Pb	Pr	Pb	Pr	Pb	Pr	Pb	Pr	Pb		
Thymol	NH	0.75	NC	0.60	NI	0.93	NM	0.99	NCy	0.89	640	4
Carvacrol	NH	0.75	NC	0.60	NI	0.96	NM	0.99	NCy	0.89	810	4
Borneol	NH	0.77	NC	0.78	NI	0.99	NM	0.98	NCy	0.88	500	4
2-Carene	NH	0.78	NC	0.71	NI	0.81	NM	0.69	NCy	0.74	4800	5
Limonene	NH	0.76	NC	0.65	NI	0.95	NM	0.97	NCy	0.82	4400	5
Camphor	NH	0.72	NC	0.68	NI	0.96	NM	0.94	NCy	0.61	775	4
Alpha-pinene	NH	0.86	NC	0.60	NI	0.99	NM	0.93	NCy	0.75	3700	5
Geraniol	NH	0.79	NC	0.76	NI	0.99	NM	0.97	NCy	0.85	2100	5
Alpha-terpinene	NH	0.78	NC	0.75	NI	0.95	NM	0.85	NCy	0.85	1680	4
Carvone	NH	0.65	NC	0.83	NI	0.99	NM	0.97	NCy	0.80	1640	4

**Table 5:** Pocket volume hierarchy of the protein targets.

Protein	Organism	Function	Top Pocket Vol (Å^3^)	VD
AF-P51764-F1	*M. aeruginosa*	Photosynthesis	812	2527
AF-W8SIR2-F1	*C. sorokiniana*	Photosynthesis	398	1252
AF-B0JFM7-F1	*M. aeruginosa*	ATP synthase	394	987
AF-W8TIQ2-F1	*C. sorokiniana*	ATP synthase	379	1000
AF-A0A2P6U0Q9-F1	*C. sorokiniana*	SOD	94	266
AF-A0A857DAR8-F1	*M. aeruginosa*	SOD	80	212

**Table 6:** Binding affinities in kcal/mol of the tested monoterpenes against key proteins from *M. aeruginosa* and *C. sorokiniana*.

Compounds	*Chlorella sorokiniana*			*Microcystis aeruginosa*		
	AF-W8TIQ2-F1	AF-A0A2P6U0Q9-F1	AF-W8SIR2-F1	AF-B0JFM7-F1	AF-A0A857DAR8-F1	AF-P51764-F1
Alpha-pinene	−5.2	−5.6	−5.3	−5.5	−4.3	−5.1
Alpha-terpinene	−5.4	−5	−5.8	−6.2	−4	−5.7
Borneol	−4.7	−4.3	−5	−4.7	−4.2	−4.6
Camphor	−4.7	−5	−4.6	−4.9	−4	−4.7
2-Carene	−5.4	−4.9	−5.9	−5.7	−4.2	−5.5
Carvacrol	−5.6	−5.2	−6	−6.2	−4.3	−5.9
Carvone	−5.5	−5.1	−6.1	−6	−4.4	−5.7
Geraniol	−5.4	−5.1	−5.3	−5.4	−4.4	−4.6
Limonene	−5.2	−4.7	−5.7	−6.1	−3.9	−5.6
Thymol	−5.9	−5	−5.9	−5.7	−4.4	−5.8

**Table 4:** In silico ecotoxicological predictions of the selected compounds using VEGA HUB QSAR models: bioconcentration factor (BCF), acute aquatic toxicity (fish, Daphnia magna, and algae), chronic no-observed-effect concentration (NOEC), and earthworm terrestrial toxicity.

	Bioaccumulation	Fish Acute Toxicity	Daphnia Toxicity	Algae Toxicity	Chronic NOEC			Terrestrial
Compounds	BCF Arnot-Gobas (log L/kg)	Fish LC_50_ Fathead Minnow EPA (mg/L)	Daphnia EC_50_ IRFMN (mg/L)	Algae EC_50_ ProtoQSAR-Combase (mg/L)	Fish NOEC IRFMN (mg/L)	Earthworm NOEC CONCERT (mg/kg)	Daphnia NOEC IRFMN (mg/L)	Algae NOEC IRFMN (mg/L)
Thymol	1.87	8.02	5.01	74.65	0.49	92.36	0.35	1.28
Carvacrol	1.86	7.54	4.05	2.24	0.30	25.69	0.25	0.83
Borneol	1.92	100.00	9.56	47.25	1.28	27.34	1.35	2.86
2-Carene	3.24	1.84	3.95	58.34	0.28	41.99	0.32	0.46
Limonene	3.02	5.15	1.10	66.18	0.27	86.51	0.17	0.67
Camphor	1.52	67.93	9.14	47.25	1.61	16.66	1.47	2.31
Alpha-pinene	3.34	25.43	1.25	53.18	0.67	64.18	0.59	0.38
Geraniol	2.44	9.81	16.09	2.73	0.38	197.07	1.19	1.81
Alpha-terpinene	3.01	5.92	1.28	66.18	0.32	48.86	0.20	0.75
Carvone	1.64	9.14	21.96	2.24	0.36	11.13	1.69	2.74

## Data Availability

The original contributions presented in this study are included in the article. Further inquiries can be directed to the corresponding author.

## Abbreviations

The following abbreviations are used in this manuscript:

MIC	Minimum inhibitory concentration
ADMET	Absorption, distribution, metabolism, excretion, and toxicity
ADME	Absorption, distribution, metabolism, and excretion
SOD	Superoxide dismutase
ATP	Adenosine triphosphate
HAB	Harmful algal bloom
DMSO	Dimethylsulfoxide
SI	Selectivity index
DDM	Disc diffusion method
TPSA	Topological polar surface area
CSP3	Fraction of carbon atoms that are sp3 hybridized
GI	Gastrointestinal absorption
BBB	Blood–brain barrier
P-gp	P-glycoprotein
NI	Non-immunotoxic
NH	Non-hepatotoxic
NM	Non-mutagenic
NC	Non-carcirogenic
NCy	Noncytotoxic
LD	Lethal dose
Pr	Prediction
Pb	Probability
VD	Volume depth
GLU	Glutamic acid
VAL	Valine
LEU	Leucine
ALA	Alanine
PHE	Phenylalanine
THR	Threonine
ASP	Aspartic acid

## Appendix A

**Table A1:** Interaction profile of the selected monoterpenes.

Compound	Category	Type	Residues	Distance
**AF-W8TIQ2-F1**				
Alpha-pinene	Hydrophobic	Pi–Sigma	A:PHE161	3.82583
	Hydrophobic	Alkyl	A:VAL319	4.6526
	Hydrophobic	Alkyl	A:LEU324	5.37171
	Hydrophobic	Alkyl	A:VAL341	3.95348
	Hydrophobic	Alkyl	A:LEU324	4.54651
	Hydrophobic	Alkyl	A:VAL341	4.94602
	Hydrophobic	Pi–Alkyl	A:PHE161	5.39542
	Hydrophobic	Pi–Alkyl	A:PHE333	5.17113
Alpha-terpinene	Electrostatic	Pi–Anion	A:GLU198	4.70266
Borneol	Hydrophobic	Alkyl	A:VAL319	4.84064
	Hydrophobic	Alkyl	A:VAL341	5.26946
Camphor	Hydrophobic	Alkyl	A:VAL319	4.99235
	Hydrophobic	Alkyl	A:LEU324	4.91277
	Hydrophobic	Alkyl	A:VAL341	5.00264
	Hydrophobic	Pi–Alkyl	A:PHE161	5.15903
2-Carene	Hydrophobic	Alkyl	A:LEU324	5.26178
	Hydrophobic	Alkyl	A:VAL341	4.41289
	Hydrophobic	Alkyl	A:VAL341	4.48138
	Hydrophobic	Pi–Alkyl	A:PHE161	4.69136
	Hydrophobic	Pi–Alkyl	A:PHE333	4.48844
Carvacrol	Electrostatic	Pi–Anion	A:GLU198	4.71272
Carvone	Hydrophobic	Alkyl	A:LEU202	4.24893
	Hydrophobic	Pi–Alkyl	A:PHE189	4.80701
Geraniol	Hydrophobic	Pi–Sigma	A:PHE161	3.68474
	Hydrophobic	Pi–Sigma	A:PHE161	3.87099
	Hydrophobic	Alkyl	A:VAL319	4.65576
	Hydrophobic	Alkyl	A:VAL341	5.01897
	Hydrophobic	Alkyl	A:VAL341	4.92738
	Hydrophobic	Alkyl	A:LEU324	4.51465
	Hydrophobic	Pi–Alkyl	A:PHE161	4.73904
	Hydrophobic	Pi–Alkyl	A:PHE333	4.90397
Limonene	Hydrophobic	Pi–Sigma	A:PHE161	3.66031
	Hydrophobic	Alkyl	A:LEU324	5.02962
	Hydrophobic	Alkyl	A:VAL341	4.54754
Thymol	Hydrogen Bond	Conventional Hydrogen Bond	A:GLU198	2.38395
	Electrostatic	Pi–Anion	A:GLU198	4.67098
**AF-A0A2P6U0Q9-F1**				
Alpha-pinene	Hydrophobic	Alkyl	A:ALA84	4.81247
	Hydrophobic	Alkyl	A:ALA87	4.31953
	Hydrophobic	Alkyl	A:ALA102	3.77355
	Hydrophobic	Alkyl	A:ALA102	5.22279
	Hydrophobic	Alkyl	A:CYS107	4.71106
	Hydrophobic	Alkyl	A:LEU83	5.16976
	Hydrophobic	Alkyl	A:LEU83	3.90185
	Hydrophobic	Pi–Alkyl	A:TRP98	4.61229
	Hydrophobic	Pi–Alkyl	A:TRP98	5.34145
	Hydrophobic	Pi–Alkyl	A:TRP98	4.52033
Alpha-terpinene	Electrostatic	Pi–Anion	A:ASP266	3.34159
	Hydrophobic	Alkyl	A:LEU79	3.90122
	Hydrophobic	Alkyl	A:VAL267	3.81675
	Hydrophobic	Pi–Alkyl	A:TRP126	4.50096
	Hydrophobic	Pi–Alkyl	A:LEU78	5.10661
	Hydrophobic	Pi–Alkyl	A:LEU268	5.1775
Borneol	Hydrogen Bond	Conventional Hydrogen Bond	A:THR27	1.82462
	Hydrophobic	Alkyl	A:LEU268	5.35196
Camphor	Hydrogen Bond	Conventional Hydrogen Bond	A:LEU108	2.38639
	Hydrophobic	Alkyl	A:LEU83	4.7911
	Hydrophobic	Alkyl	A:ALA84	5.39438
	Hydrophobic	Alkyl	A:ALA87	5.30964
	Hydrophobic	Alkyl	A:ALA102	4.5031
	Hydrophobic	Pi–Alkyl	A:TRP98	4.92108
2-Carene	Hydrophobic	Alkyl	A:ALA84	4.88649
	Hydrophobic	Alkyl	A:ALA87	3.72969
	Hydrophobic	Alkyl	A:ALA102	4.19335
	Hydrophobic	Alkyl	A:ALA102	3.9422
	Hydrophobic	Alkyl	A:LEU108	5.13035
	Hydrophobic	Alkyl	A:LEU108	4.38127
	Hydrophobic	Pi–Alkyl	A:TRP98	4.44577
	Hydrophobic	Pi–Alkyl	A:TRP98	5.08184
Carvacrol	Electrostatic	Pi–Anion	A:ASP266	3.30899
	Hydrophobic	Pi–Alkyl	A:LEU78	5.1062
	Hydrophobic	Pi–Alkyl	A:LEU268	5.14725
Carvone	Hydrogen Bond	Conventional Hydrogen Bond	A:LEU79	2.30701
	Hydrophobic	Alkyl	A:LEU268	5.48166
	Hydrophobic	Pi–Alkyl	A:PHE26	4.77183
Geraniol	Hydrogen Bond	Conventional Hydrogen Bond	A:THR27	1.86167
	Hydrogen Bond	Conventional Hydrogen Bond	A:THR27	2.87127
	Hydrophobic	Alkyl	A:LEU78	5.35246
	Hydrophobic	Alkyl	A:LEU268	4.57329
	Hydrophobic	Alkyl	A:LEU78	5.14306
	Hydrophobic	Alkyl	A:LEU79	5.34879
	Hydrophobic	Alkyl	A:LEU79	4.60268
	Hydrophobic	Pi–Alkyl	A:PHE26	5.38454
Limonene	Hydrophobic	Alkyl	A:LEU79	4.46069
	Hydrophobic	Alkyl	A:LEU268	5.20642
	Hydrophobic	Pi–Alkyl	A:PHE26	4.75452
Thymol	Electrostatic	Pi–Anion	A:ASP266	3.3133
	Hydrophobic	Alkyl	A:LEU79	3.93332
	Hydrophobic	Alkyl	A:VAL267	3.82213
	Hydrophobic	Pi–Alkyl	A:TRP126	4.54136
	Hydrophobic	Pi–Alkyl	A:LEU78	5.08598
	Hydrophobic	Pi–Alkyl	A:LEU268	5.13351
**AF-W8SIR2-F1**				
Alpha-pinene	Hydrophobic	Alkyl	A:VAL185	4.35943
	Hydrophobic	Alkyl	A:VAL185	5.22038
	Hydrophobic	Alkyl	A:ILE60	5.42991
	Hydrophobic	Alkyl	A:VAL185	3.87392
Alpha-terpinene	Hydrophobic	Pi–Sigma	A:ASP170	3.96172
	Hydrophobic	Alkyl	A:ILE60	4.54392
	Hydrophobic	Pi–Alkyl	A:VAL185	4.7068
Borneol	Hydrogen Bond	Conventional Hydrogen Bond	A:ASP170	2.52283
	Hydrophobic	Alkyl	A:VAL185	4.08215
Camphor	Hydrophobic	Alkyl	A:VAL330	5.37104
2-Carene	Hydrophobic	Alkyl	A:PRO173	5.48042
	Hydrophobic	Alkyl	A:VAL185	4.80936
	Hydrophobic	Alkyl	A:VAL185	3.83259
	Hydrophobic	Alkyl	A:ILE60	4.60806
	Hydrophobic	Pi–Alkyl	A:TYR161	5.45032
	Hydrophobic	Pi–Alkyl	A:PHE182	4.2301
Carvacrol	Hydrogen Bond	Conventional Hydrogen Bond	A:GLU333	2.28057
	Hydrophobic	Alkyl	A:ILE60	4.66468
	Hydrophobic	Pi–Alkyl	A:VAL185	4.47244
Carvone	Hydrophobic	Alkyl	A:VAL185	4.41102
	Hydrophobic	Pi–Alkyl	A:TYR161	5.26407
Geraniol	Hydrophobic	Alkyl	A:VAL185	4.35412
	Hydrophobic	Alkyl	A:ILE60	4.60358
	Hydrophobic	Alkyl	A:VAL185	4.40599
Limonene	Hydrophobic	Alkyl	A:VAL185	4.40158
	Hydrophobic	Alkyl	A:ILE60	4.55297
Thymol	Hydrogen Bond	Conventional Hydrogen Bond	A:GLU333	2.11502
	Hydrophobic	Pi–Sigma	A:VAL185	3.88023
	Hydrophobic	Alkyl	A:VAL185	4.1544
	Hydrophobic	Pi–Alkyl	A:TYR161	4.71925
	Hydrophobic	Pi–Alkyl	A:PHE182	4.48039
**AF-B0JFM7-F1**				
Alpha-pinene	Hydrophobic	Pi–Sigma	A:TYR351	3.56289
	Hydrophobic	Alkyl	A:VAL169	3.87154
	Hydrophobic	Alkyl	A:ALA427	4.90695
	Hydrophobic	Alkyl	A:ALA427	4.90931
	Hydrophobic	Alkyl	A:ALA427	3.6979
	Hydrophobic	Alkyl	A:VAL169	4.5487
	Hydrophobic	Pi–Alkyl	A:TYR351	3.99179
	Hydrophobic	Pi–Alkyl	A:TYR351	5.075
	Hydrophobic	Pi–Alkyl	A:PHE424	4.86422
Alpha-terpinene	Hydrophobic	Pi–Sigma	A:PHE424	3.81306
	Hydrophobic	Pi-Pi Stacked	A:TYR351	3.77216
	Hydrophobic	Pi–Alkyl	A:TYR351	4.16501
	Hydrophobic	Pi–Alkyl	A:VAL169	4.91261
	Hydrophobic	Pi–Alkyl	A:ALA427	4.78861
Borneol	Hydrophobic	Pi–Sigma	A:TYR351	3.66669
	Hydrophobic	Alkyl	A:VAL169	4.59659
	Hydrophobic	Alkyl	A:ALA427	4.64045
Camphor	Hydrophobic	Alkyl	A:VAL169	4.45407
	Hydrophobic	Alkyl	A:ALA427	4.60132
	Hydrophobic	Pi–Alkyl	A:TYR351	4.46316
2-Carene	Hydrophobic	Alkyl	A:VAL169	4.70323
	Hydrophobic	Alkyl	A:ALA427	4.52413
	Hydrophobic	Alkyl	A:ALA427	3.70714
	Hydrophobic	Alkyl	A:VAL169	4.80331
	Hydrophobic	Pi–Alkyl	A:TYR351	3.91747
	Hydrophobic	Pi–Alkyl	A:PHE424	4.14824
Carvacrol	Hydrophobic	Pi–Sigma	A:PHE424	3.73167
	Hydrophobic	Pi-Pi Stacked	A:TYR351	3.78764
	Hydrophobic	Pi–Alkyl	A:TYR351	4.05429
	Hydrophobic	Pi–Alkyl	A:VAL169	5.01546
	Hydrophobic	Pi–Alkyl	A:ALA427	4.72782
Carvone	Hydrophobic	Pi–Sigma	A:PHE424	3.73015
	Hydrophobic	Alkyl	A:VAL169	5.38911
	Hydrophobic	Alkyl	A:ALA427	3.46232
	Hydrophobic	Pi–Alkyl	A:TYR351	5.42058
	Hydrophobic	Pi–Alkyl	A:PHE424	5.20301
Geraniol	Hydrophobic	Alkyl	A:VAL169	4.03247
	Hydrophobic	Alkyl	A:ALA427	4.46194
	Hydrophobic	Alkyl	A:VAL169	4.8575
	Hydrophobic	Pi–Alkyl	A:TYR351	4.09936
	Hydrophobic	Pi–Alkyl	A:PHE430	4.03414
Limonene	Hydrophobic	Pi–Sigma	A:PHE424	3.74769
	Hydrophobic	Alkyl	A:VAL169	4.9179
	Hydrophobic	Alkyl	A:ALA427	4.72748
	Hydrophobic	Pi–Alkyl	A:TYR351	3.80916
	Hydrophobic	Pi–Alkyl	A:TYR351	4.22519
Thymol	Hydrophobic	Pi-Pi Stacked	A:TYR351	3.77669
	Hydrophobic	Pi–Alkyl	A:TYR351	4.32038
	Hydrophobic	Pi–Alkyl	A:ALA427	4.85413
**AF-A0A857DAR8-F1**				
Alpha-pinene	Hydrophobic	Alkyl	A:LEU53	4.40441
	Hydrophobic	Pi–Alkyl	A:TYR3	4.99164
	Hydrophobic	Pi–Alkyl	A:TYR39	4.96756
Alpha-terpinene	Hydrophobic	Pi-Pi T-shaped	A:TYR3	4.79404
Borneol	Hydrogen Bond	Conventional Hydrogen Bond	A:MET1	2.02991
	Hydrophobic	Alkyl	A:MET1	5.31473
Camphor	Hydrogen Bond	Conventional Hydrogen Bond	A:LEU53	2.66137
2-Carene	Hydrophobic	Alkyl	A:MET1	3.72117
	Hydrophobic	Alkyl	A:LEU53	4.23483
	Hydrophobic	Pi–Alkyl	A:TYR3	5.36683
	Hydrophobic	Pi–Alkyl	A:TYR3	4.9517
	Hydrophobic	Pi–Alkyl	A:TYR39	4.86403
Carvacrol	Hydrogen Bond	Conventional Hydrogen Bond	A:LEU53	2.25782
	Hydrogen Bond	Conventional Hydrogen Bond	A:GLU54	2.5313
	Hydrophobic	Pi-Pi T-shaped	A:TYR3	4.83965
Carvone	Hydrogen Bond	Conventional Hydrogen Bond	A:THR4	2.22738
	Hydrophobic	Pi–Alkyl	A:TYR3	5.4234
	Hydrophobic	Pi–Alkyl	A:TYR39	5.38934
Geraniol	Hydrogen Bond	Conventional Hydrogen Bond	A:LEU51	2.14096
	Hydrophobic	Alkyl	A:LEU53	5.38031
	Hydrophobic	Alkyl	A:LEU53	5.40451
	Hydrophobic	Alkyl	A:LEU53	4.71342
	Hydrophobic	Pi–Alkyl	A:TYR3	5.05212
	Hydrophobic	Pi–Alkyl	A:TYR3	5.10134
	Hydrophobic	Pi–Alkyl	A:TYR3	5.30612
	Hydrophobic	Pi–Alkyl	A:TYR3	4.81626
	Hydrophobic	Pi–Alkyl	A:TRP77	4.81611
Limonene	Hydrophobic	Alkyl	A:ALA2	4.56113
	Hydrophobic	Alkyl	A:LYS44	3.70772
	Hydrophobic	Alkyl	A:LYS44	4.59728
Thymol	Hydrogen Bond	Conventional Hydrogen Bond	A:ASP49	2.8153
	Hydrophobic	Alkyl	A:ALA2	4.03577
	Hydrophobic	Alkyl	A:LYS44	4.93372
	Hydrophobic	Pi–Alkyl	A:ALA2	4.75925
	Hydrophobic	Pi–Alkyl	A:LYS44	3.69554
**AF-P51764-F1**				
Alpha-pinene	Hydrophobic	Pi–Sigma	A:PHE186	3.71843
	Hydrophobic	Alkyl	A:MET183	4.81706
	Hydrophobic	Alkyl	A:VAL290	4.64729
	Hydrophobic	Alkyl	A:ILE192	5.07305
	Hydrophobic	Alkyl	A:MET183	4.39399
	Hydrophobic	Alkyl	A:ILE192	3.98788
	Hydrophobic	Pi–Alkyl	A:PHE182	4.81204
	Hydrophobic	Pi–Alkyl	A:PHE186	4.51843
Alpha-terpinene	Hydrophobic	Alkyl	A:ILE60	4.53546
	Hydrophobic	Alkyl	A:PRO173	4.72405
	Hydrophobic	Pi–Alkyl	A:VAL185	4.73449
Borneol	Hydrogen Bond	Conventional Hydrogen Bond	A:ASN181	2.72073
	Hydrophobic	Alkyl	A:MET328	5.15076
Camphor	Hydrogen Bond	Conventional Hydrogen Bond	A:ASN338	2.23474
	Hydrogen Bond	Conventional Hydrogen Bond	A:PHE339	2.12574
2-Carene	Hydrophobic	Alkyl	A:MET183	4.74226
	Hydrophobic	Alkyl	A:ILE192	5.10888
	Hydrophobic	Alkyl	A:VAL157	5.25582
	Hydrophobic	Alkyl	A:VAL290	4.97824
	Hydrophobic	Alkyl	A:MET183	3.8311
	Hydrophobic	Alkyl	A:ILE192	4.12869
	Hydrophobic	Pi–Alkyl	A:PHE182	5.34041
	Hydrophobic	Pi–Alkyl	A:PHE186	5.04807
Carvacrol	Hydrophobic	Pi–Sigma	A:ASP170	3.9579
	Hydrophobic	Alkyl	A:ILE60	4.52577
	Hydrophobic	Pi–Alkyl	A:VAL185	4.50583
Carvone	Hydrophobic	Alkyl	A:ILE60	4.47799
Geraniol	Hydrogen Bond	Conventional Hydrogen Bond	A:ALA68	3.08547
	Hydrogen Bond	Carbon Hydrogen Bond	A:SER79	3.75583
	Hydrophobic	Alkyl	A:VAL67	4.79085
	Hydrophobic	Alkyl	A:ALA81	4.0078
	Hydrophobic	Alkyl	A:VAL83	4.79155
	Hydrophobic	Alkyl	A:ILE60	4.01788
	Hydrophobic	Alkyl	A:VAL67	4.00393
Limonene	Hydrophobic	Alkyl	A:VAL185	4.56299
	Hydrophobic	Alkyl	A:ILE60	4.60899
	Hydrophobic	Alkyl	A:PRO173	4.65796
Thymol	Hydrophobic	Alkyl	A:VAL185	4.1074
	Hydrophobic	Pi–Alkyl	A:TYR161	4.87178
	Hydrophobic	Pi–Alkyl	A:PHE182	4.26292
	Hydrophobic	Pi–Alkyl	A:VAL185	4.22521
